# Intratumoral Bacteria are Uncommon in Gastrointestinal Stromal Tumor

**DOI:** 10.1245/s10434-024-16526-9

**Published:** 2024-11-22

**Authors:** Katherine J. Tardy, Hyunjee V. Kwak, Andrew D. Tieniber, Alina K. Mangold, Juan E. Perez, Kevin Do, Shan Zeng, Ferdinando Rossi, Ronald P. DeMatteo

**Affiliations:** Department of Surgery, Perelman School of Medicine, University of Pennsylvania, Philadelphia, PA USA

## Abstract

**Background:**

Gastrointestinal stromal tumor (GIST) is the most common human sarcoma with over 5000 new patients diagnosed in the USA each year. The tumor originates from the interstitial cells of Cajal and forms an intramural lesion most commonly in the stomach or small intestine. The gut microbiome has been linked to other gastrointestinal cancers and a recent paper purported that GISTs contain substantial intratumoral bacteria. The purpose of this study is to further evaluate the presence of bacteria in GISTs.

**Patients and Methods:**

We collected 25 tumor samples of varying size and location from 24 patients under sterile conditions in the operating room immediately following surgical resection. 16S quantitative polymerase chain reaction (qPCR) and 16S ribosomal RNA (rRNA) gene amplicon sequencing were performed to evaluate the bacterial species present in each tumor. Retrospective chart review was performed to determine tumor characteristics, including tumor size, location, imatinib exposure, and mucosal involvement.

**Results:**

In 23 of the 25 tumor samples, there were fewer than 100 copy numbers of 16S rRNA per uL, indicating an absence of a significant bacterial load. 16S rRNA gene amplicon sequencing of the remaining two samples, one gastric tumor and one duodenal tumor, revealed the presence of normal intestinal bacteria. These two tumors, along with three others, had disruption of the mucosal lining.

**Conclusions:**

GISTs generally lack substantial bacteria, except in some cases when the tumor disrupts the mucosa.

Gastrointestinal stromal tumor (GIST) is the most common human sarcoma with over 5000 new cases diagnosed in the USA each year.^1^ The tumor originates from the interstitial cells of Cajal and forms a submucosal lesion, most commonly in the stomach or small intestine. Up to 85% of tumors develop owing to mutations in the genes encoding the receptor tyrosine kinases *KIT* or *PDGFRA*. A minority of GISTs disrupt the mucosal lining of the stomach or small intestine and can cause occult gastrointestinal bleeding or intratumoral infection.

Microbes are involved in the development of cancer and the host immune response to cancer cells, but few directly cause cancer.^2^ For example, colibactin, the first metabolite implicated in cancer development, is produced by intestinal *Escherichia coli* and causes DNA damage and chromosomal instability, leading to the development of colorectal cancer.^3^ Similarly, in other gastrointestinal cancers, mucosal microbial dysbiosis and loss of diversity has been shown to contribute to carcinogenesis. The microbiome further influences the treatment response of multiple types of cancers. For example, the intestinal microbes *Lactobacillus johnsonii* and *Enterococcus hirae* alter the efficacy of cyclophosphamide in melanoma and sarcoma models.^4^ In addition, metabolism by intratumoral *Gammaproteobacteria* decreases the effects of gemcitabine therapy in pancreatic cancer by directly inactivating the drug.^5^ In addition to traditional chemotherapy, differential responses to immunotherapy have also been linked to the microbiome. In melanoma, various gut bacterial species affect the body’s immune responses to anti-cytotoxic T-lymphocyte associated protein 4 (CTLA4) and anti-programmed cell death protein 1 (PD-1) therapy.^6,7^

There is one publication of the microbiome in human GISTs. Ravegnini et al. reported that more than 60% of GISTs contain a detectable bacterial load.^8^ Further, GIST exhibited a loss of bacterial diversity as well as altered metabolic pathways as the tumor progressed from microGIST < 1 cm to classic GIST > 1 cm. Given these findings, we sought to further investigate bacteria within GISTs.

## Patients and Methods

### Specimen Collection

We collected 25 tumor samples from 24 patients with primary or metastatic GIST who underwent surgery at our institution between 3 March 2020 and 25 October 2022 and gave written consent for participation in an institutional review board (IRB)-approved protocol at the University of Pennsylvania (IRB protocol 829796). Immediately after the tumor was removed from the patient, a 1–2cm intratumoral portion was excised with sterile instruments in the operating room. The biopsies were inserted into sterile plastic containers, immediately flash frozen in liquid nitrogen, and stored at −80 °C.

### qPCR and Gene Amplicon Sequencing

Tumor specimens were sent to the Microbiome Center of the Children’s Hospital of Philadelphia for processing on 16 November 2022. Total DNA was extracted from the frozen tumor biopsies using the Qiagen Power Soil Pro Kit, automated on Qiacube. Libraries were generated by using barcoded PCR primers that bind to the V1-V2 region of the 16s rRNA gene. qPCR was performed in duplicate using Q5 High-Fidelity DNA Polymerase (NEB, Ipswich, MA) in which samples were treated as low-biomass (diluted 1:20) and run for 40 cycles alongside two extract controls, two negative PCR controls and one positive PCR control. Overall, 12 standards of known concentration were used to generate a standard curve including 11 positive control standards and 1 standard blank. The 16S-specific primers BSF8 and BSR357 were used, respectively. Following qPCR, Ct values and final copy number per uL were derived. 16S rRNA gene amplicon sequencing was performed on tumor samples with a sufficient bacterial load, defined as copy number over 100 copies per uL, to evaluate the bacterial species present. Sequencing was performed on the Illumina MiSeq using 2 × 250 bp chemistry. Negative controls during the extraction, library preparation, and sequencing steps included DNA-free water, an empty well, and a blank swab. A taxonomic heat map was generated in R using package ggplot2.

### Clinical Chart Review

The authors of this study did have access to patient identifiers, and retrospective chart review was performed to determine tumor characteristics, including tumor size, location, imatinib exposure, preoperative antibiotics for surgical site infection (SSI) prophylaxis, race/ethnicity, and mucosal involvement as defined by preoperative evidence of ulceration or bleeding or the presence of mucosal invasion in the final surgical specimen. Plots of clinical data were generated in Prism. The small number of positive samples precluded statistical analysis with sufficient power to assess clinical correlation.

## Results

Samples were collected from eight primary gastric tumors, six primary small intestinal tumors, four metastatic liver tumors, and seven metastatic peritoneal tumors. Tumor size varied from 1.1 to 17.5 cm on pathologic analysis. There was evidence of preoperative mucosal communication in 5 of the 14 patients with gastric or intestinal GIST. The tumors were stratified according to treatment response at the time of surgery based on serial radiologic imaging as untreated (*n* = 11), imatinib-sensitive (*n* = 7), or imatinib-resistant (*n* = 7) as shown in Table 1. Copy numbers of 16S rRNA per uL was less than 100 in 23 of the 25 tumor samples, indicating an absence of a significant bacterial load. The remaining two samples, one untreated primary gastric tumor and one untreated primary duodenal tumor, had mucosal ulceration with 230 and 837 copies of the gene encoding 16S rRNA per uL DNA, respectively (Table 1).

**Table 1:** Tumor features and 16S rRNA copy number per uL DNA in human GISTs

Tumor number	Treatment status	Tumor location	Tumor size (cm)	Ethnicity	Mucosal involvement	Copy per uL DNA	Mutational status
1	Untreated	Stomach	5.1	White/non-Hispanic	+	836.8	Kit Exon 11
2	Untreated	Stomach	11.1	Black/African American	+	74.2	Kit Exon 11
3	Untreated	Stomach	13.5	White/non-Hispanic	−	44.5	PDGFRA D842V
4	Untreated	Stomach	6.8	Not-reported	+	12.8	Kit Exon 11
5	Untreated	Small bowel	17.5	Black/African American	−	10.3	Wild-type
6	Untreated	Small bowel	6.2	White/non-Hispanic	−	9.6	Kit Exon 11
7	Untreated	Small bowel	2.8	Black/African American	+	229.9	Kit Exon 11
8	Untreated	Small bowel	2.0	White/non-Hispanic	−	12.3	Kit Exon 11
9	Untreated	Small bowel	6.8	White/non-Hispanic	−	47.3	Kit Exon 11
10	Untreated	Peritoneum	7.1	White/non-Hispanic	n/a^a^	6.7	SDH Deficient
11	Untreated	Peritoneum	3.8	White/non-Hispanic	n/a	12.8	Kit Exon 9
12	Sensitive	Stomach	11.1	White/non-Hispanic	−	15.1	Kit Exon 11
13	Sensitive	Stomach	15.1	White/non-Hispanic	−	0.0	Kit Exon 11
14	Sensitive	Stomach	6.4	Black/African American	+	0.0	Kit Exon 11
15	Sensitive	Stomach	8.8	White/non-Hispanic	−	42.8	Kit Exon 11
16	Sensitive	Small bowel	2.0	White/non-Hispanic	−	11.0	Kit Exon 11
17	Sensitive	Liver	1.6	White/non-Hispanic	n/a	7.6	Kit Exon 11
18	Sensitive	Liver	9.3	White/non-Hispanic	n/a	9.0	Kit Exon 11
19	Resistant	Liver	5.2	White/non-Hispanic	n/a	7.0	Kit Exon 9
20	Resistant	Liver	5.0	White/non-Hispanic	n/a	6.5	Kit Exon 11
21	Resistant	Peritoneum	1.1	White/non-Hispanic	n/a	81.9	Not Tested
22	Resistant	Peritoneum	8.0	White/non-Hispanic	n/a	4.0	Kit Exon 11
23	Resistant	Peritoneum	6.6	White/non-Hispanic	n/a	15.6	Kit Exon 9
24	Resistant	Peritoneum	2.0	White/non-Hispanic	n/a	7.9	Kit Exon 9
25	Resistant	Peritoneum	2.6	Not-reported	n/a	14.2	Kit Exon 11

^a^n/a, Not applicable

**Fig. 1:**
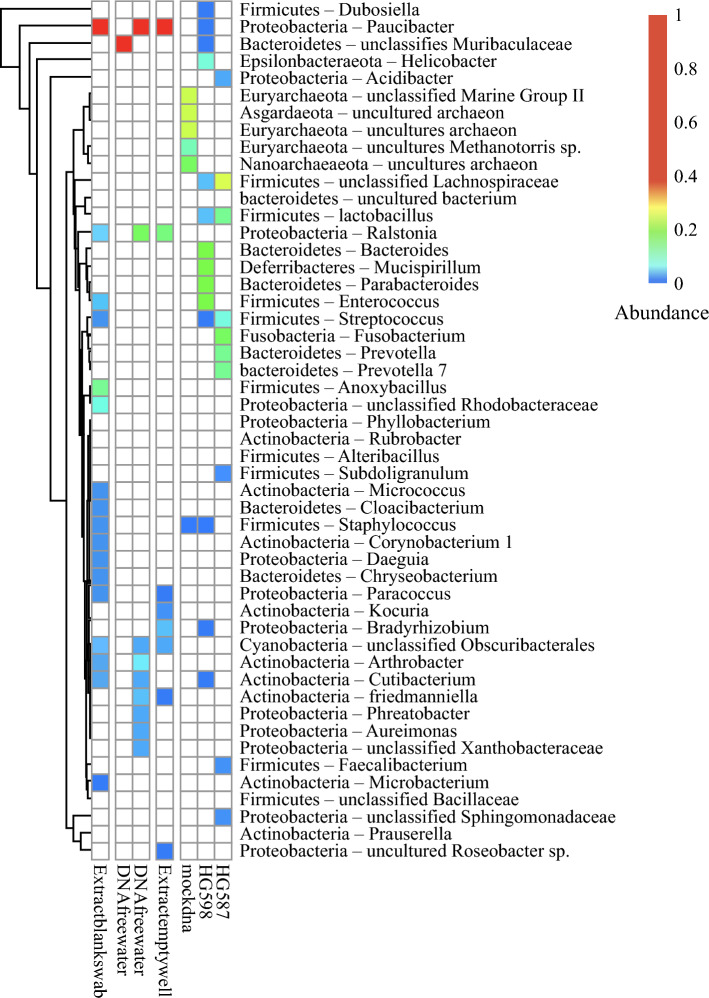
Bacterial species in human GISTs. Taxonomic heatmap showing relative abundance of bacterial species in human GISTs with red indicating the highest abundance

**Fig. 2:**
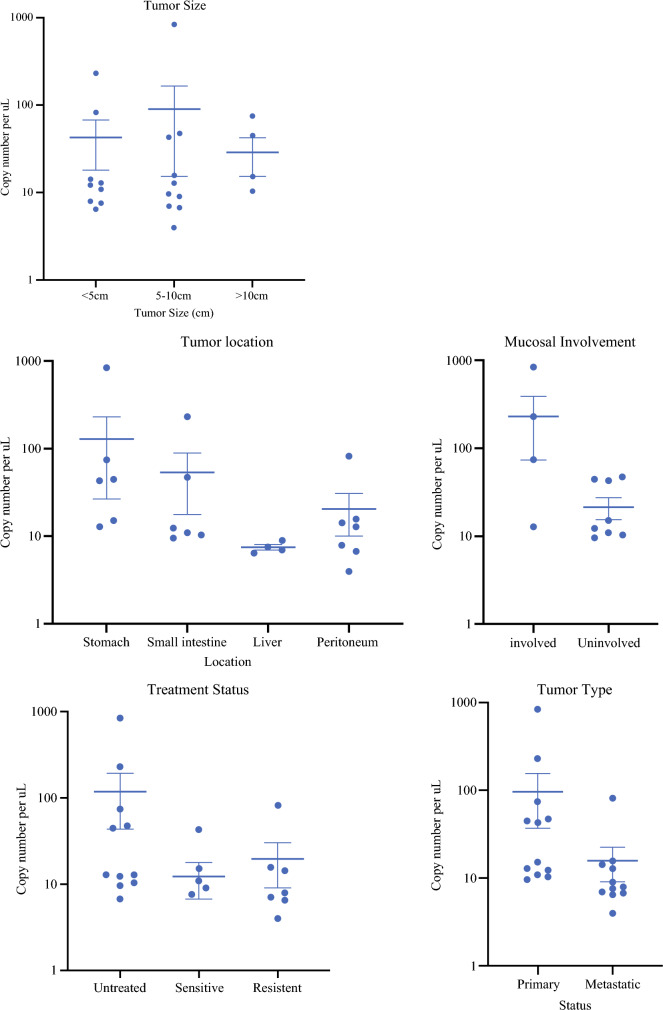
Clinical characteristics of human GISTs. Tumor size, location, preoperative mucosal involvement, treatment status, tumor type, and bacterial load in human GISTs

16S rRNA gene amplicon sequencing revealed the presence of multiple bacterial species in these two specimens. Any species present in the extract controls or negative PCR controls were excluded as were species present at very low abundance as defined by an abundance value less than 0.2. Further analysis confirmed the presence of multiple intestinal bacterial species including *Lachnospiraceae*, *Lactobacillus*, *Bacteroides*, *Mucispirilium*, *Parabacteroides*, *Enterococcus*, *Fusobacterium*, and *Prevotella* (Fig. 1). There was no apparent relationship between bacterial load and tumor size, location, or treatment status. Two of the five tumors with mucosal communication contained a significant amount of bacteria (Fig. 2).

## Discussion

It is now clear that the microbiome is important in human disease, including gastrointestinal cancers such as gastric and colorectal adenocarcinoma. Wen et al. reviewed the non-*Helicobacter pylori* gastric microbes involved in gastric carcinogenesis.^9^ *Proteobacteria, Firmicutes*, *Bacteroidetes*, *Actinobacteria*, and *Fusobacteria* were the most abundant phyla consistently observed in gastric biopsies from healthy patients. Microbial dysbiosis leading to decreased microbial diversity and increased oral and intestinal opportunistic pathogens in the gastric mucosa was associated with gastric carcinogenesis. Similarly, the gut microbiome in colorectal cancer not only influences oncogenesis but also the therapeutic response to treatment with chemotherapy or immunotherapy. Wong, et al. reported that dysregulation of the gut microbiota is involved in colon cancer tumorigenesis.^10^ However, these tumors differ from GISTs in that they arise from the mucosa.

We did not find evidence of significant intratumoral bacteria in GIST. This is in contrast to the findings of Ravegnini et al., who reported more than 60% of GISTs contain bacteria from the phylum *Proteobacteria* and genus *Prevotella*.^8^ GIST is an intramural tumor but can sometimes ulcerate through the mucosal lining of the gastrointestinal tract. We found that 5 of 14 gastric and intestinal tumors disrupted the mucosa. In the few samples where we found substantial bacteria, the most abundant bacterial genera included *Lachnospiraceae*, *Lactobacillus*, *Bacteroides*, *Mucispirilium*, *Parabacteroides*, *Enterococcus*, *Fusobacterium*, and *Prevotella*, many of which comprise the normal intestinal microbiome.^9,11–13^ Importantly, these bacteria were only found in primary gastric or intestinal tumors with evidence of mucosal disruption. We found no evidence of bacteria in any GIST metastases in the liver or peritoneum.

There are several potential explanations for the discrepancy in our findings. Ravegnini et al. did not specify whether their tumors were surgical specimens or biopsies, but presumably the larger lesions were surgical specimens. The manuscript states the microGISTs were identified during investigational procedures but does not mention how tissue samples were obtained particularly for microGIST that do not typically require surgical resection. Transmucosal endoscopic biopsy could introduce mucosal bacteria into a submucosal lesion. More importantly, their specimens were paraffin-embedded whereas ours were freshly procured from the operating room under sterile conditions. While the authors attempted to control for potential contamination by sampling the edge of the paraffin wax for the presence of bacteria, there is still the possibility of contamination during the process of specimen embedding. Another methodologic difference that may account for the discrepancy in our findings is our cutoff of 100 copy numbers per uL. This cutoff was chosen on the basis of the standard curve generated at the time of PCR amplification. Samples above this threshold contained sufficient material to sequence, whereas samples below required reamplification to sequence the sample. Sequencing of these reamplified samples revealed a low abundance of a wide range of bacteria, which we interpreted as too low to confer clinical significance. This cutoff relies on the assumption that microbial burden correlates with clinical significance and is a limitation of our study. Additionally, we did not include rectal GISTs to focus on the most common primary tumor sites of GIST, the stomach and small intestine. Rectal tumors are exposed to colonic flora which may result in an entirely different tumor microenvironment. We also did not examine fungi, including yeasts, which have roles in human cancers.^14,15^ For example, *Saccharomyces paradoxus* was associated with an increased response to anti-PD-1 immunotherapy in melanoma, while increased levels of *Malassezia restricta* and* Candida albicans* were linked with an increased risk of progression and worse response to anti-PD-1 treatment.^15^

Normal intestinal flora can have a protective or pathogenic role in the development of human disease. Two bacterial genera in our samples, *Mucispirilium* and *Parabacteroides*, are particularly interesting as they have been associated with both protection against and the development of various human diseases, including gastrointestinal diseases and cancer. *Mucispirilium* has been associated with inflammatory conditions in the intestine. In immunodeficient mice, *M*. *schaedleri* was causally linked to the development of Crohn’s-like colitis.^16^ However, in an immune-competent host, this same species protects against non-typhoidal *Salmonella enterica serovar Typhimurium* (S. Tm) colitis.^17^ Meanwhile, the relative abundance of *Parabacteroides* has been associated with multiple human diseases, such as obesity, inflammatory bowel disease, and metabolic syndrome.^18^ In some cases, *Parabacteroides* may promote host health by regulating the host immune system and metabolism, relieving inflammation, and secreting metabolites. Importantly, *Parabacteroides* has not been associated with the development of cancer, and some species have even been reported as protective against colon tumorigenesis.^19^ While we have identified several abnormal bacterial species present in GIST surgical specimens, cancer-related roles for these species have not yet been discovered.

## Conclusion

GISTs from the stomach and small bowel without mucosal involvement have a minimal amount of intratumoral bacteria. Some GISTs with mucosal disruption have bacteria that contain local flora.
